# Feeding exogenous dsRNA interferes with endogenous sRNA accumulation in *Paramecium*

**DOI:** 10.1093/dnares/dsaa005

**Published:** 2020-04-27

**Authors:** Sivarajan Karunanithi, Vidya Oruganti, Raphael de Wijn, Franziska Drews, Miriam Cheaib, Karl Nordström, Martin Simon, Marcel H Schulz

**Affiliations:** 1 Cluster of Excellence for Multimodal Computing and Interaction, and Department for Computational Biology & Applied Algorithms, Max Planck Institute for Informatics, Saarland Informatics Campus, Saarbrücken, Germany; 2 Graduate School of Computer Science, Saarland Informatics Campus, Saarland University, Saarbrücken, Germany; 3 Institute for Cardiovascular Regeneration, Goethe University Hospital, Frankfurt am Main, Germany; 4 Molecular Cell Dynamics, Centre for Human and Molecular Biology, Saarland University, Saarbrücken, Germany; 5 Molecular Cell Biology and Microbiology, Wuppertal University, Wuppertal, Germany; 6 Genetics/Epigenetics, Centre for Human and Molecular Biology, Saarland University, Saarbrücken, Germany

**Keywords:** environmental RNAi, dsRNA feeding, siRNA, off-target

## Abstract

Supply of exogenous dsRNA (exo-dsRNA), either by injection or by feeding, is a fast and powerful alternative to classical knockout studies. The biotechnical potential of feeding techniques is evident from the numerous studies focusing on oral administration of dsRNA to control pests and viral infection in crops/animal farming. We aimed to dissect the direct and indirect effects of exo-dsRNA feeding on the endogenous short interfering RNA (endo-siRNA) populations of the free-living ciliate *Paramecium*. We introduced dsRNA fragments against Dicer1 (*DCR1*), involved in RNA interference (RNAi) against exo- and few endo-siRNAs, and an RNAi unrelated gene, *ND169*. Any feeding, even the control dsRNA, diminishes genome wide the accumulation of endo-siRNAs and mRNAs. This cannot be explained by direct off-target effects and suggests mechanistic overlaps of the exo- and endo-RNAi mechanisms. Nevertheless, we observe a stronger down-regulation of mRNAs in *DCR1* feeding compared with *ND169* knockdown. This is likely due to the direct involvement of *DCR1* in endo-siRNA accumulation. We further observed a *cis*-regulatory effect on mRNAs that overlap with phased endo-siRNAs. This interference of exo-dsRNA with endo-siRNAs warrants further investigations into secondary effects in target species/consumers, risk assessment of dsRNA feeding applications, and environmental pollution with dsRNA.

## 1. Introduction

The term environmental RNA interference (RNAi) refers to a mechanism in which cells or species can take up regulatory RNA from the medium, food or the environment.^1^ Two surprising findings contribute to this phenomenon: RNA can be taken up into cells systemically distributed among multicellular organisms. Second, the foreign RNA can be processed into small RNA with regulatory power in gene regulation, thus environmental RNA is capable of interfering in gene expression.

dsRNA uptake mechanisms have been intensively studied in the nematode *Caenorhabditis elegans*, where two RNA transporters have been identified. Sid-2 imports dsRNA from the gut-lumen into cells, and Sid-1 is necessary for systemic RNAi, i.e. to transport short interfering RNAs (siRNAs) from cell to cell.^2^^,^^3^ Although the Sid-1 channel is widely distributed in many but not all eukaryotes, a systematic analysis of Sid-1 positive species for being capable of systemic RNAi is missing. One reason for that is probably Sid-deficient nematode species also shows systemic RNAi.^4^ As a result, there seem to be many undiscovered mechanisms for species to realize environmental RNAi.

Nevertheless, this system seems to be quite attractive for biotechnology: dsRNA could be seen as a drug and easily delivered to cells and organisms to control, e.g. virus replication. In addition to the initial studies in *C. elegans*, many studies have investigated the oral administration of bacterial-, plant- or *in vitro-*transcribed dsRNA to shrimp,^5^^,^^6^ planarians,^7^ and insects^8^ making significant progress in the usage of dsRNA as a species-specific drug for pest control. However, little is known about off-target effects and a suitable risk assessment for artificial dsRNA in the environment, in genetic engineered plants or in animal farming.

We therefore need to understand more about individual RNA uptake and individual siRNA accumulation pathways of different species. The issue becomes even more important as cells and species usually do not only show a single but also several RNAi mechanisms occurring in parallel. Often different small RNA species, e.g. siRNA, micro-RNA (miRNA), or piwi-interacting RNA (piRNA) are involved. Moreover, these mechanisms are not independent, but share individual components. Dicer for instance, responsible for sRNA cleavage from dsRNA, has been shown to be involved in many different mechanisms, e.g. *C. elegans* Dcr-1.^9^ Cleavage of these dsRNA occurring in equal intervals is called phasing.^10^ Importantly there is not only an overlap between different siRNA mechanisms but also to the miRNAs. Feeding of exogenous dsRNA (exo-dsRNA), and subsequent siRNA accumulation, was shown to increase transcript levels of miRNAs targets, thus implicating a competition between environmental and endogenous RNAi on the siRNA and miRNA level.^11^

We used *Paramecium tetraurelia*, a single-celled free-living genetic model to analyse the effect of dsRNA feeding on the endogenous siRNA (endo-siRNA) population and endogenous gene regulation. This organism is capable of RNAi by feeding dsRNA producing bacteria^12^ and the underlying mechanisms have been intensively studied. Two RNA-dependent RNA polymerases (RDRs) and *DCR1* are necessary for primary siRNA production, but the genetic requirements for secondary siRNA products are less clear.^13–15^ A recent genome wide study identified 2,602 endo-siRNA producing loci. Of these, 1,618 endo-siRNA loci overlap with annotated genes in different transcriptomic states (serotypes) of *Paramecium*.^16^ This study also revealed that many endo-siRNA loci depend on the two RDRs (RDR1 and RDR2), which are involved in the feeding pathway, too. This may indicate that there could be a mechanistic overlap between endo- and exo-RNAi. *Paramecium* does not show any canonical miRNAs. In contrast to other organisms, *Paramecium’s* endo-siRNAs are not strictly associated with gene silencing, because many highly expressed genes show high abundance of siRNAs as well.^16^ These may be involved *in trans* silencing processes or they might be the result of unspecific accumulation such as spurious Dicer activity or inefficient siRNA degradation mechanisms.

In this study, RNAi was applied to two different serotypes cultivated at different temperatures to analyse the phenomena in different backgrounds, because serotypes differ not only in the expression of the individual serotype gene but also large parts of their transcriptome.^17^ Moreover, some small RNA pathways in *Paramecium* also show a temperature dependency, as transgene induced silencing of the *ND169* gene works most efficiently at high temperatures (31°C).^18^ Using RNAi by feeding, we introduced dsRNA against the major Dicer gene, *DCR1*, and a control gene, *ND169*. We investigated the effect of exo-dsRNA to the accumulation of the recently identified 1,618 endo-siRNAs overlapping with annotated genes, in different transcriptomic states (serotypes) in *Paramecium*. We aimed to dissect the genetic requirements of phased and non-phased endo-siRNAs as well as to analyse these for potential genome wide off-target effects by deep sequencing of siRNAs and mRNA.

## 2. Materials and methods

### 2.1. Cell culture and RNAi

Serotype pure cultures of *P. tetraurelia* stock 51 were cultivated under standard conditions using *Klebsiella planticola* infused WGP (wheat grass powder) medium [wild-type (WT) cultures]. Serotype 51A cultures were kept at 31°C, 51B at 24°C, and checked for surface antigen expression by immobilization with polyclonal antibodies as described.^17^ RNAi by feeding was carried out as previously described using *Escherichia coli*.^12^^,^^19^ The feeding fragments used for dsRNA synthesis had the following genomic positions (kind gift of E. Meyer, Paris): scaffold51_70:312063-313251 for *DCR1* (PTET.51.1.G0700179) and scaffold51_21:137857-138267 for *ND169* (PTET.51.1.G0210080).

### 2.2. RNA isolation and sequencing

Total RNA was isolated from vegetative cells (autogamy was checked by nuclei staining with DAPI) using Tri-Reagent (Sigma) as described^20^ before. After additional DNAse digestion and subsequent purification with acid phenol, sRNA fractions were enriched by denaturing gel electrophoresis and cutting the gel from 17 to 25 nts. After re-isolation of the sRNAs by extraction in 0.3 M NaCl, sRNAs were precipitated and we used the NEB Small RNA library preparation Kit (New England Biolabs) with elongated 3´-adapter ligation to limit biases against 2´-O-methylated siRNAs. Long RNA libraries were prepared after poly-A enrichment using the NEBNext Ultra directional RNA preparation Kit (New England Biolabs). Both setups were sequenced on a HiSeq2500 (Illumina), sRNAs in Rapid mode and long RNA in High Output mode. Reads were trimmed for adapters and low-quality bases by the cutadapt (version 1.4.1^21^) wrapper trim galore (version 0.3.3; https://www.bioinformatics.babraham.ac.uk/projects/trim_galore/, accessed 28 April 2020).

### 2.3. Data description

We utilized the sRNA-seq replicates of WT serotype 51A, and 51B, which we obtained from our recent study^16^ (Cluster definition data; ENA Accession: PRJEB25903). Further we performed sRNA-seq on RNAi knockdown samples (two replicates each for both 51A, and 51B serotypes). We obtained mRNA expression data for WT serotypes (51A, 51B) produced as part of our earlier study^17^ (ENA Accession: PRJEB9464). Additionally, we sequenced mRNA from RNAi knockdown samples (three replicates each for 51A, and 51B serotypes). All RNAi knockdown sequencing datasets produced for this study can be accessed at ENA (Accession: PRJEB33364).

### 2.4. Quantification of small RNA

We pre-processed the sRNA datasets to represent only 21-25 nt sRNA reads in this study. We retrieved the locations of the 1,618 endo-siRNA loci, which overlap with annotated genes from the supplementary methods of Karunanithi et al.,^16^ and quantified them using the RAPID software.^22^ We utilized the default parameters of RAPID, which performs error-free alignments using bowtie2,^23^ while allowing multi-mapping reads.

### 2.5. Normalization of small RNA data

We performed the knockdown corrected scaling (KDCS) normalization method^22^ implemented in RAPID to normalize the sRNA read counts. In a nutshell, the KDCS method subtracts the reads aligning to the feeding associated regions from the estimated read library size before performing a total count scaling.

Let us assume that we want to normalize the reads for an endo-siRNA locus with a read count of *R*. The total number of reads in the respective library mapping to the genome is *T*, with *K* number of feeding associated reads. We define the normalized read abundance of the endo-siRNA region, *R*′, as R′=R×M/(T-K), where M is the maximum of the values $\left(T_{1}-K_{1}),..,(T_{n}-K_{n}\right)$ for *n* samples.

**Figure 1:**
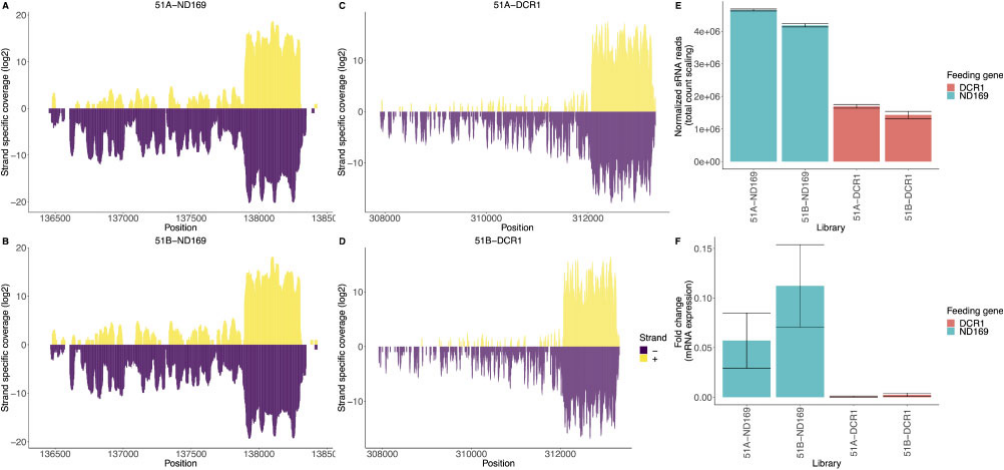
Strand-specific small RNA coverage (*y*-axis; log2) of the genes *ND169* (A and B), *DCR1* (C and D) in the respective serotype-knockdown library is shown. (E) A bar plot showing the normalized sRNA read counts (*y*-axis) of the knocked down RNAi genes in the respective serotype-knockdown samples (*x*-axis; library). Just for this figure element, normalization was carried out using only total count scaling (TCS), i.e. knockdown-associated regions were not removed. (F) Fold change of gene expression (TPM in knockdown vs. TPM of wild-type of mRNAs targeted by primary siRNAs in both serotypes).

All sRNA data normalization in this work is done using the KDCS method, except for Fig. 1E. As we want to show the abundance of the feeding associated reads in Fig. 1E, we correct for changes in total sequence depth (total count scaling) but do not correct for small RNA reads from the feeding region. Under the assumptions described earlier, we perform total count scaling as R′=R×M/T where M is the maximum of the values $T_{1},..,T_{n}$ of all *n* samples.

### 2.6. Quantification of mRNA expression

We used Salmon^24^ (version 0.8.2) to quantify the mRNA expression in transcripts per million (TPM), with default parameters. All quantifications were done using the recent annotation^25^ of *P. tetraurelia* MAC genome (version 2; stock 51). Mean of replicates were used for all analyses, unless otherwise mentioned. While calculating the expression of the knockdown genes in Fig. 1F, we excluded the feeding-associated regions from the transcripts for the respective RNAi knockdown samples. To account for alignment artifacts, we added 100 bps both upstream and downstream, while excluding the feeding-associated regions.

### 2.7. Clustering of expression data

We created heatmaps of the endo-siRNA and mRNA expression data using the heatmap.2 function from the R/Bioconductor package gplots (version 3.0.1.1). We performed a hierarchical clustering of the libraries with complete linkage using an euclidean distance measure.

### 2.8. Differential expression analysis

Utilizing the WT libraries as control samples, we performed a differential expression (DE) analysis of endo-siRNAs raw read counts for each RNAi library. The R/Bioconductor package DESeq2 (version 1.18.1)^26^ was used to perform the DE analysis. We filtered for differentially expressed endo-siRNAs with a Benjamini–Hochberg’s^27^ multiple testing corrected *P*-value <0.05 and subjected them for downstream analyses. For the DE analysis of mRNAs, we subjected the raw read counts obtained from HTSeq (version 0.9.0)^28^ to the DESeq2 package and used the same cutoffs as for siRNAs.

### 2.9. Off-target analysis

We created all possible 23-mers from the feeding regions of both *ND169* and *DCR1* genes, as well as their reverse complement 23-mers. We aligned these 23-mers against the rest of the *P. tetraurelia* MAC genome (version 2; stock 51). The bowtie2^23^ aligner was used to perform local alignments (- -local) and report up to 100 distinct alignments for each read (−*k* 100). Further, we identified the genes overlapping with a unique exact match from these alignments.

### 2.10. GO enrichment analysis

We performed gene ontology (GO) enrichment analysis using Ontologizer (version 2.0).^29^ We utilized the default options of the Ontologizer tool except for setting the options to Benjamini–Hochberg method for multiple testing corrections. GO terms with a corrected *P*-value < 0.05 are considered statistically significant. All *Paramecium* genes were used as the population set.

## 3. Results and discussion

### 3.1. Analysis of feeding associated siRNAs of *DCR1* and *ND169*

Two genes were silenced by RNAi and this was carried out at two different transcriptomic backgrounds (serotypes) at 31°C for cells expressing serotype 51A and at 24°C for cells expressing serotype 51B. We have chosen *DCR1* as it was shown to be involved in transgene-induced silencing as well as dsRNA feeding, which both accumulate 23 nt siRNAs.^14^^,^^20^ 23 nt is also the pre-dominant length of endo-siRNAs.^16^ As a control, we used dsRNA feeding against a gene involved in trichocyst discharge (*ND169*), which has no known relation with siRNA accumulation.

After RNA isolation and sequencing, we first analysed the siRNAs associated with dsRNA feeding. Fig. 1A–D shows coverage plots of the two feeding genes. We can observe the accumulation of primary siRNAs in the feeding regions and a significantly lower amount of secondary siRNAs outside this region. We can confirm that these siRNAs are an effect of feeding, as the siRNAs of these two feeding genes in WT serotypes have low coverage (Supplementary Fig. S1). As the coverage plots represent raw reads, the bar plot in Fig. 1E shows normalized data for the feeding associated siRNAs. *DCR1* feeding shows a lower amount of siRNAs compared with *ND169* feeding. As *DCR1* has been shown to be involved in the feeding pathway,^13^^,^^30^ silencing of *DCR1* by feeding could inhibit its own silencing. This form of recursive RNAi could be the reason why we observe lower abundance of primary siRNAs in *DCR1* feeding. However, Fig. 1F shows the knockdown efficiency of the target genes by displaying the reduction in fold changes. Both, *DCR1* and *ND169* silencing lead to mRNA reduction greater than 95%, with *DCR1* silencing being particularly efficient.

### 3.2. Endogenous siRNAs in control feeding are not WT

**Figure 2:**
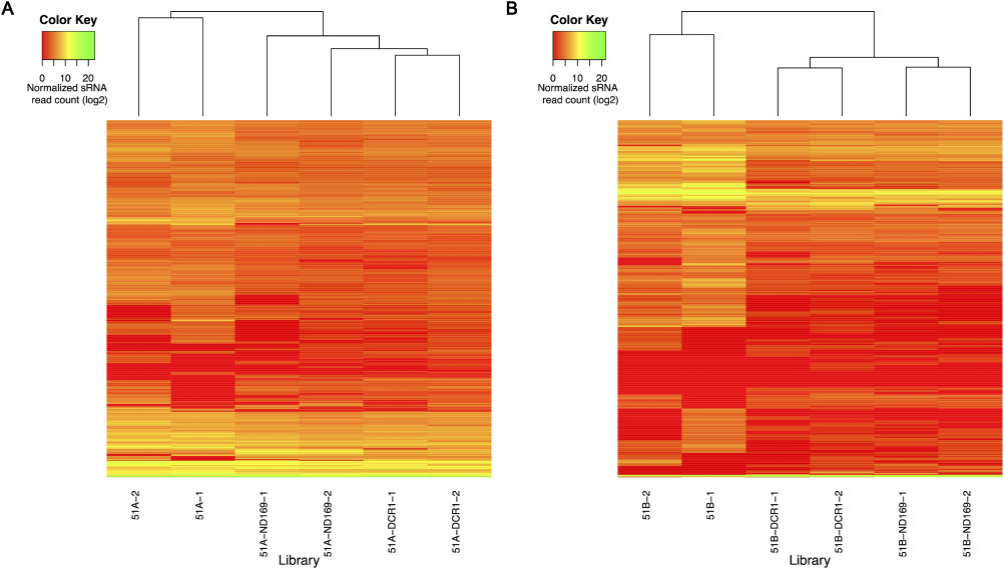
Heatmap of normalized sRNA read counts after hierarchical clustering (Euclidean distance measure) of the 1,618 endo-siRNAs (rows) for all replicates (columns) respective to the serotype 51A (A) and 51B (B) is shown.

We next analysed the endo-siRNAs of the feeding cultures and compared them to WT cells of the respective serotype fed with regular and non-dsRNA producing bacteria. We took advantage of the recently described genome wide analysis of endo-siRNA producing loci in the vegetative genome of *P. tetraurelia*, which identified 1,618 endo-siRNA loci overlapping with gene annotations in different serotypes in *Paramecium*.^16^ To analyse sRNA abundance in these loci for the different feeding experiments, we normalized the read data using the KDCS method implemented in the RAPID pipeline.^22^ The KDCS method scales the read counts of each endo-siRNA in a library to the library with highest read counts, after eliminating the feeding associated siRNAs which are usually highly abundant (see Materials and methods). We utilized these normalized read counts of the endo-siRNA loci of all libraries to investigate the clustering pattern of replicates, specific to each serotype (Fig. 2A and B). We noted that WT samples clearly separated from all feeding cultures. It is surprising as the *ND169* gene is thought not to be involved in siRNA pathways, we expected *ND169* feeding replicates to cluster with WT replicates. We note that WT and feeding cultures differ in the bacterial strains used as food (*K. planticola* and *E. coli*, respectively). However, as both belong to the Enterobacteriaceae family and as *Paramecium* shows almost identical division rates (data not shown), it is unlikely that differences in the observed siRNA composition are due to the food bacteria.

**Figure 3:**
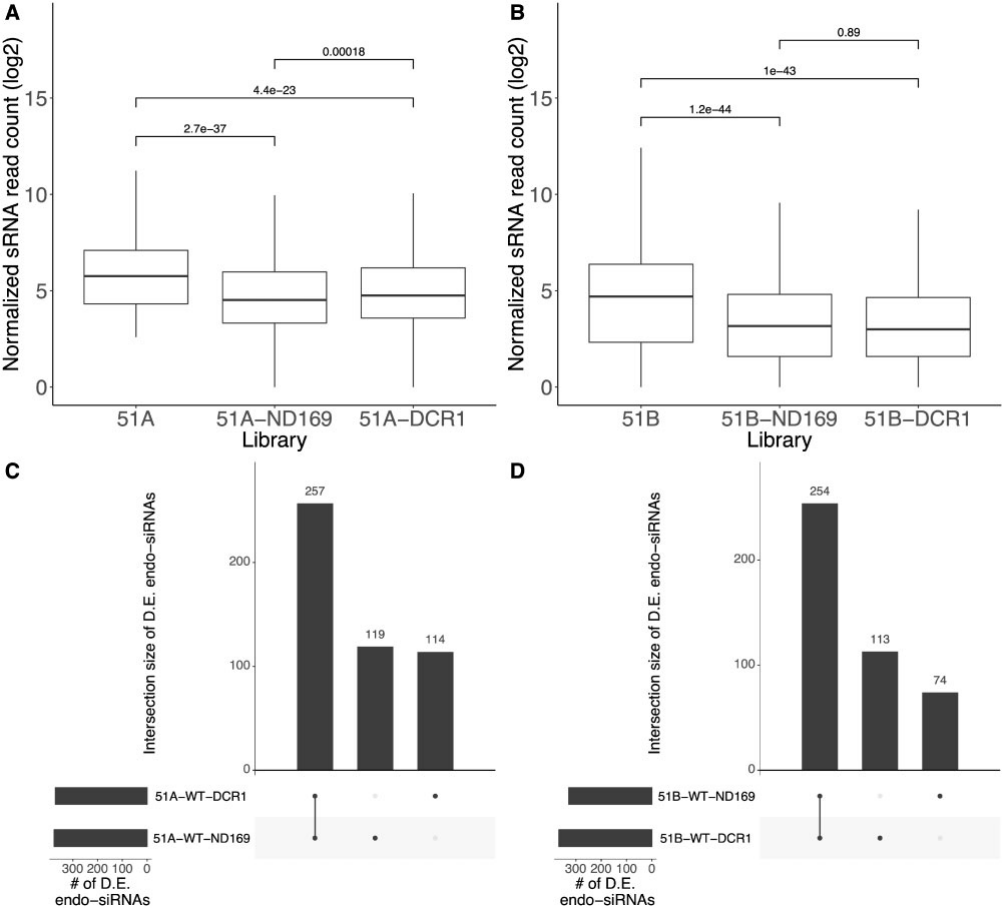
Quantification and differential expression of the 1,618 endo-siRNAs. (A and B) Boxplots of the 1,618 normalized endo-siRNA read counts (*y*-axis; log2) of serotype (51A and 51B, respectively) and their knockdowns (ND169 and DCR1). (C and D) Set intersection plots of differentially expressed endo-siRNA clusters in the knockdown libraries against each wild-type library for 51A, and 51B, respectively.

To get more insight into the differences of end-siRNAs we investigated their abundance distribution. Figure 3A and B shows boxplots visualizing the abundance of endo-siRNAs in all experiments for the two serotypes (replicates were merged). A statistically significant reduction (Wilcoxon test *P*-value < 0.05) of siRNA abundance is apparent in each feeding culture compared with the WTs without dsRNA diet. In both serotypes, the endo-siRNA accumulation pattern indicates *ND169* feeding to be closer to *DCR1* silencing than to WTs suggesting that *ND169* silencing does not solely affect the *ND169* mRNA.

We performed a DE analysis of the endo-siRNAs between the WT and the feeding samples. Figure 3C and D shows a set intersection plot (UpSetR plot^31^) of the significantly differentially expressed endo-siRNAs (see Materials and methods; Supplementary Table S1), for the two serotypes 51A and 51B, respectively. In serotypes 51A and 51B, the *DCR1* feeding samples have 371 and 367 differentially expressed endo-siRNAs, respectively. Of them approximately 70% of the endo-siRNAs (257 in 51A and 254 in 51B) are differentially expressed in *ND169* feeding as well, which suggest a common response to exo-dsRNA.

We performed a GO enrichment analysis, using the Ontologizer software, to investigate whether genes associated with the differentially expressed endo-siRNAs have any over-represented GO terms (Supplementary Table S1). We identified diverse functions and processes associated with these genes. Following are some of the significantly enriched terms: *cofactor metabolic process*, *Pteridine-containing compound metabolic process*, *single-multicellular organism process*, *multicellular organism process*, *developmental process*, and others. These results suggest that feeding interferes with a diverse set of pathways irrespective of the feeding gene.

Our results are the first evidence that application of exo-dsRNA alters endo-siRNA accumulation at large scale in *Paramecium*. One reason could be that exo-dsRNA could have off-targets. We discuss these off-targets in relation with gene expression in the next section. Another reason could be that the massive amounts of exo-dsRNA saturate molecular components of the feeding pathway. Hence, those components which are additionally involved in endogenous regulation of gene expression have lower capacity for their endogenous role. This implies that cell cultures undergoing dsRNA feeding should not be considered WT, at least on the siRNA level.

### 3.3. Feeding of dsRNA causes de-regulation of gene expression

**Figure 4:**
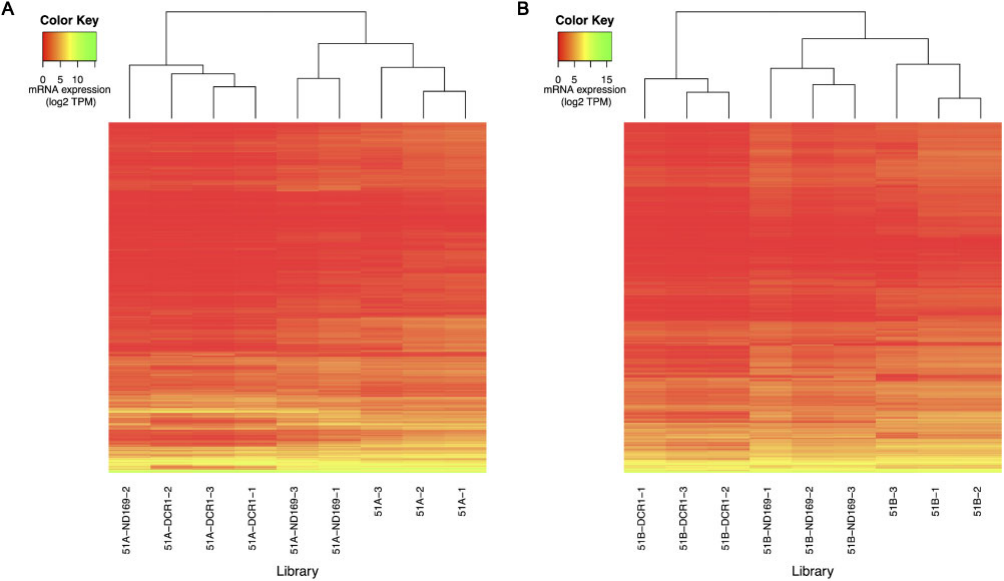
Heatmap of mRNA expression after hierarchical clustering (Euclidean distance measure) of all the mRNAs (rows) for all replicates (columns) respective to the serotype 51A (A) and 51B (B) is shown.

Due to the loss of endo-siRNAs in all feeding experiments, we investigated whether this is accompanied by changes in gene expression. We prepared poly-A enriched libraries for WT and RNAi against two genes, *DCR1* and as control *ND169*. The mRNA libraries of RNAi samples were generated from the respective biological sample used for sRNA libraries. We quantified the gene expression of all the annotated genes of *P. tetraurelia* using the Salmon software (see Materials and methods). For each serotype, we created a heatmap of the gene expression values (Fig. 4A and B) showing the clustering of replicates. We can observe that WT samples cluster separately in both serotypes. Similar to the analysis of siRNAs (Fig. 2A and B), we observe that there are large changes in the mRNA transcriptome after *ND169* and *DCR1* feeding. However, the *ND169* feeding replicates (except one replicate in serotype 51A) are relatively closer to the WT replicates than what we observed in endo-siRNA accumulation, according to our clustering analysis.

**Figure 5:**
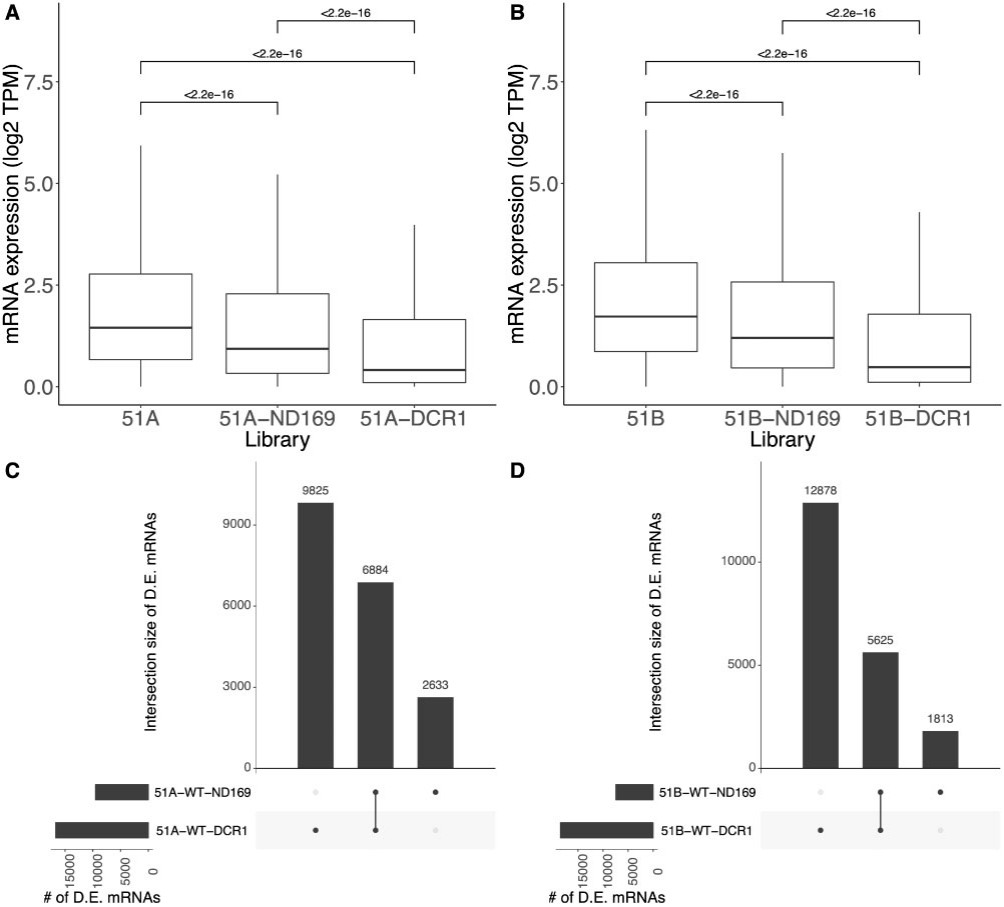
(A and B) Boxplots of genome-wide mRNA expression (*y*-axis; TPM) of serotype (51A and 51B, respectively) and their knockdowns (ND169 and DCR1). Reads mapping to the feeding-associated regions was removed prior to expression quantification. (C and D) Set intersection plots of differentially expressed (D.E.) mRNA in the knockdown libraries against each wild-type library for 51A, and 51B, respectively.

To analyse the differences in mRNA expression in more detail, we first checked the distribution of gene expression (mean of the replicates), visualizing them as boxplots shown in Fig. 5A and B. We observe a statistically significant reduction in the gene expression in the feeding experiments in both serotypes (Wilcoxon test *P*-value < 0.05). However, unlike the endo-siRNA accumulation (Fig. 3A and B), we can observe a statistically significant difference between the *ND169* control feeding and the *DCR1* feeding as well in both serotypes. This asserts the observed clustering pattern in Fig. 4. Albeit observing a rather equal amount of endo-siRNA loss in both feeding samples, the loss in mRNA is different among them, with stronger reduction of mRNA expression in *DCR1* feeding. One possibility for this behaviour is that many of the produced endo-siRNAs are either spurious or *cis* inactive Dicer products. It is unclear whether these spurious or *cis* inactive Dicer products have a different regulatory capacity. However, our earlier work has shown that endo-siRNAs do not cause *cis* silencing effects, for many loci.^16^

Subsequently, we performed differential gene expression analysis between the WT, and feeding samples (see Materials and methods; Supplementary Table S1). Figure 5C and D shows set intersection plots of the significantly differentially expressed genes (see Materials and methods) for serotypes 51A and 51B, respectively. In both serotypes, *DCR1* feeding has the highest and unique set of differentially expressed genes. However, approximately 30–40% of the differentially expressed genes in *DCR1* are commonly found in the *ND169* control feeding as well. It seems likely that genes, which are uniquely differentially expressed in *DCR1*, are due to direct effects of *DCR1* being involved in endo-siRNA accumulation. In contrast, commonly differentially expressed genes are probably de-regulated as a response to the feeding process rather than the causal effect of the knocked-down gene. Supplementary Fig. S2 shows the MA plots of the up and down-regulated genes in each feeding sample against the WT serotype.

GO enrichment analysis of these commonly differentially expressed genes reveals diverse sets of biological processes such as *nucleoside phosphate metabolic process, gene expression, biosynthetic processes, ATPase activity*, and *proteolysis*. These results indicate that feeding affects a diverse set of pathways, which seem to be involved in the general depletion of endo-siRNAs and mRNAs that we observe.

### 3.4. Could direct off-target effects cause transcriptomic changes?

Another possibility would be that the feeding constructs we used have unintended off-targets triggering a cascade of transcriptomic alterations. We created all possible 23 bp sub-sequences of the feeding-region (see Materials and methods) and aligned them to the rest of the genome. We found five and two exact matching off-target genes for *ND169* and *DCR1* feeding regions, respectively. The DE status of these genes in the respective library is shown in Supplementary Table S1. Only some of the off-targets are differentially expressed in some of the libraries. With these data we can cautiously conclude that the large number of differentially expressed genes are unlikely to be an off-target effect, but a general response to the massive dsRNA feeding. We want to exercise caution in interpreting this off-target analysis, because a study on *Drosophila* shows that the sRNA fit to the target must not be over the full length. The Piwi Aubergine targets RNA for slicing with complementarity to the loaded sRNA only from nucleotides 2-16.^32^ Of course, the shorter the complementary sequence, the more off-target effects could occur. Although, it is unlikely as this would cause a massive cross silencing in the transcriptome, especially if we include 2° siRNAs in this scenario. However, in our off-target analysis we do not observe any 2-16 nt alignments.

In our case one of the most likely explanations seems to be an overload of RNAi components by exo-dsRNA, which will then have less capacity for their function in endo-siRNA biogenesis. In addition, we do not see strong regulation of RNAi components in feeding conditions, which could contribute to altered siRNA accumulation. Supplementary Fig. S3 shows the regulation of RNAi components of *Paramecium* and most of them show indeed a slight down-regulation similar to the entire transcriptome. Among several up and down-regulated RNAi components, the only known feeding component showing a slight up-regulation is RDR2, an RNA-dependent RNA polymerase. RDR2 is known to be involved in feeding and endo-siRNA accumulation.^15^^,^^16^.

Our data about feeding mechanisms in *Paramecium* document the aberrations of endo-siRNAs and transcriptome for the first time, but our current data do not allow us to decide whether off-target effects are sequence dependent or caused by more indirect effects.

### 3.5. Phased endo-siRNAs show alterations in gene expression *in cis*

Further, we dissected the endo-siRNAs between loci showing phased siRNAs and such without. Phasing is a process, where long dsRNA precursors are cut into equidistant siRNAs by Dicer or Dicer-like enzymes. However, phased siRNAs are not exclusively due to Dicer cleavage. For instance, piRNA/Ago can also cut a ssRNA into phased siRNAs.^33^ Among the 1,618 endo-siRNA producing loci in the vegetative genome of *P. tetraurelia*, 81 and 66 endo-siRNA loci were characterized as phased in serotypes 51A and 51B, respectively.^16^

**Figure 6:**
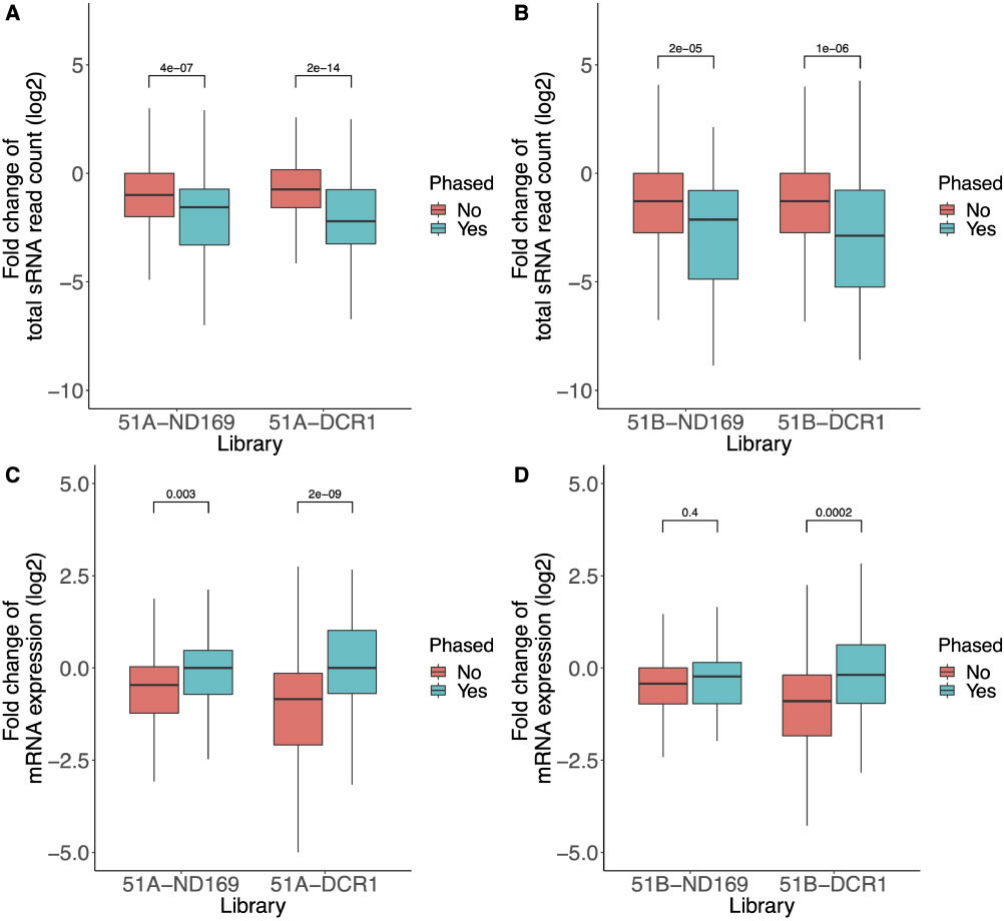
Dissecting the 1,618 endo-siRNA producing loci depending on their phasing prediction. (A and B) Fold change of the endo-siRNAs read count (*y*-axis, log2 knockdown/wild type), categorized into phased and unphased loci, is shown for knockdown libraries of serotype 51A, and 51B, respectively. (C and D) Fold change of mRNA expression (*y*-axis, log2 knockdown/wild type), only for the genes associated with the endo-siRNAs categorized into phased and unphased loci, is shown for the knockdowns of serotype 51A and 51B, respectively. *P*-Values in all plots are from two-tailed Wilcoxon significance tests.

Figure 6A and B shows the fold change of endo siRNA reads in the feeding sample against the WT. We can observe a statistically significant reduction of phased siRNAs compared with non-phased ones (Wilcoxon test *P*-value < 0.05) in all samples. In both serotypes, the strongest reduction of phased endo siRNAs can be found in *DCR1* silencing. This may fit the involvement of this enzyme in dsRNA feeding and transgene-induced silencing, both of which depend on massive accumulation of phased siRNAs.^14^^,^^20^ As the fold change interpretation (Fig. 6) neglects the absolute abundance, we show in Supplementary Fig. 4 that the abundance of phased endo-siRNAs are higher than the unphased.

We consequently asked for changes of gene expression of these loci and dissected genes into two groups with and without overlaps to phased endo-siRNAs. There were 68, and 48 genes overlapping with the phased endo-siRNAs in serotype 51A and 51B, respectively. Figure 6C and D shows the fold change of mRNA expression in RNAi samples against the WT samples of 51A, and 51B, respectively. We observe a statistically significant difference in mRNA fold changes between the phased and unphased loci in all feeding experiments, except for *ND169* feeding in serotype 51B. In other words, genes with unphased endo-siRNAs show reduced expression, whereas many genes with phased endo-siRNAs show more stable mRNA expression compared with WT.

In contrast, in our previous work we found that genes overlapping phased endo-siRNA loci showed increased mRNA expression after knockout of RDR2.^16^ However, we do not observe such an increase in mRNA expression in our feeding experiments. Our data do not allow us to dissect why the loss of endo-siRNA in *ND169* feeding of serotype 51B (Fig. 6D) does not cause altered gene expression for phased endo-siRNAs. We do not detect any significant difference neither in sense/antisense bias nor read length distribution (Supplementary Figs 5 and 6).

## 4. Conclusions

In the past, many studies used RNAi by feeding and also recursive RNAi experiments which were successful in *Paramecium,* and many other organisms, in the identification of RNAi pathway components. However, usually these approaches used individual reporter genes rather than genome-wide transcriptomic approaches. Our data show that RNAi by feeding causes genome-wide alterations in both the endo-siRNAs and mRNA levels. However, this seems not to be associated with drastic alterations of gene expression at least in *cis*.

Researchers using the feeding technique to study the gene expression caused by RNAi must exercise caution. One must distinguish between the gene expression changes by the genes involved in the feeding pathway and the ones which are merely a response to the feeding technique. The overlap of differentially expressed genes between the control *ND169* and *DCR1* feeding shown in Fig. 5C and D emphasizes the importance of choosing an appropriate control and using an unrelated gene for silencing seems still the appropriate control whether for a normal knockdown or for recursive RNAi.

Our observed endo-siRNA, and mRNA expression changes in *Paramecium,* due to exo-dsRNA application, complement a recent set of studies that show a large diversity of different off-target effects in other species. Recent evidence reports off-target effects in *C. elegans* in three different ways including the rescue of mir-35-41 triggered temperature sensitive reduction in progeny viability.^34^ As we were not able to predict any miRNAs in *Paramecium*,^16^ this clearly differs and suggests species-specific off-target effects, which cannot be generalized. A similar study has recently reported that even in mammalian cells a higher overlap between dsRNA induced and miRNA pathways are expected.^35^ The question remains whether it is true that the high abundance of dsRNA in most feeding approaches is solely responsible for sequence independent off-target effects. It was shown in *Paramecium*, that even ssRNA from food bacteria (rRNA; mRNA) becomes converted into siRNAs by the feeding pathway.^14^ One could assume that the food in general, not necessarily dsRNA engineered food, could affect endo-siRNA composition.

All of this basic knowledge, which is still fragmented, needs to be considered in biotechnological approaches. An increasing number of studies uses dsRNA produced by bacteria or plants to induce virus resistance or pest-lethality by targeting individual genes on the sequence level. This adds to the discussion whether dsRNA application in the field needs to be considered a genetic manipulation or maybe even an epigenetic manipulation. Usually, RNA-treated organisms are considered GMO-free. Especially in plants, direct application of RNA is very efficient and can be used for trans species silencing of genes in insects, mites and nematodes.^36^ When introducing massive amounts of RNA in field studies, one should expect more genome-wide effects than those induced by simple homology-dependent silencing of one individual locus.

## Supplementary Material

dsaa005_Supplementary_Data

## Accession numbers

BCWF01000001-BCWF01000044
