# Supplementary material for: Feeding exogenous dsRNA interferes with endogenous sRNA accumulation in Paramecium
Source: DNA Res. 2020 Apr 27;27(1):dsaa005. doi: 10.1093/dnares/dsaa005 (PMC7315353; doi:10.1093/dnares/dsaa005)
Supplement: dsaa005_Supplementary_Data [file dsaa005_supplementary_data.zip › Supp/SuppFigures.pdf]

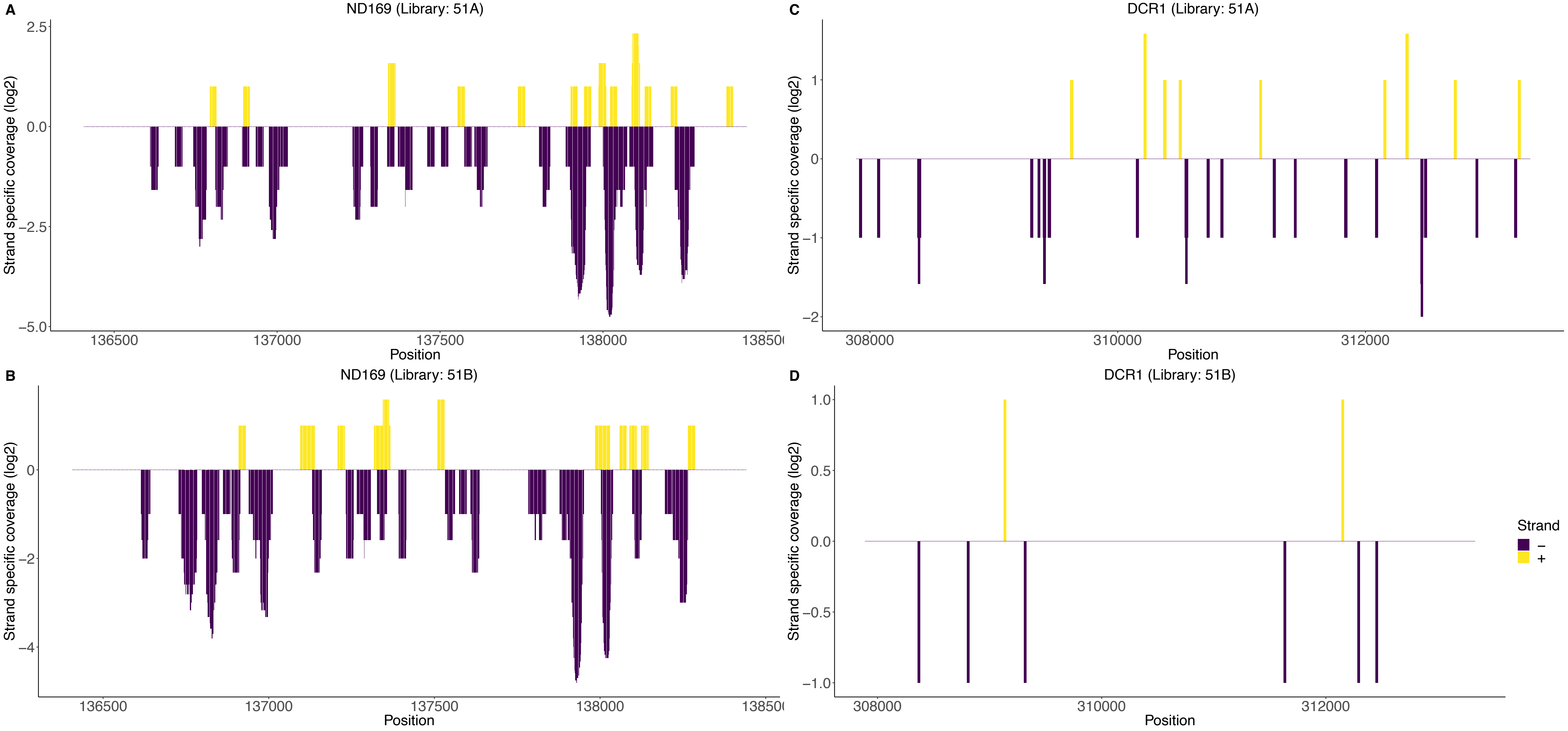

Supplementary Figure 1: Strand specific small RNA coverage (y-axis; log2) of the genes ND169 (A,B), DCR1(C,D) in the wildtype serotype libraries are shown.

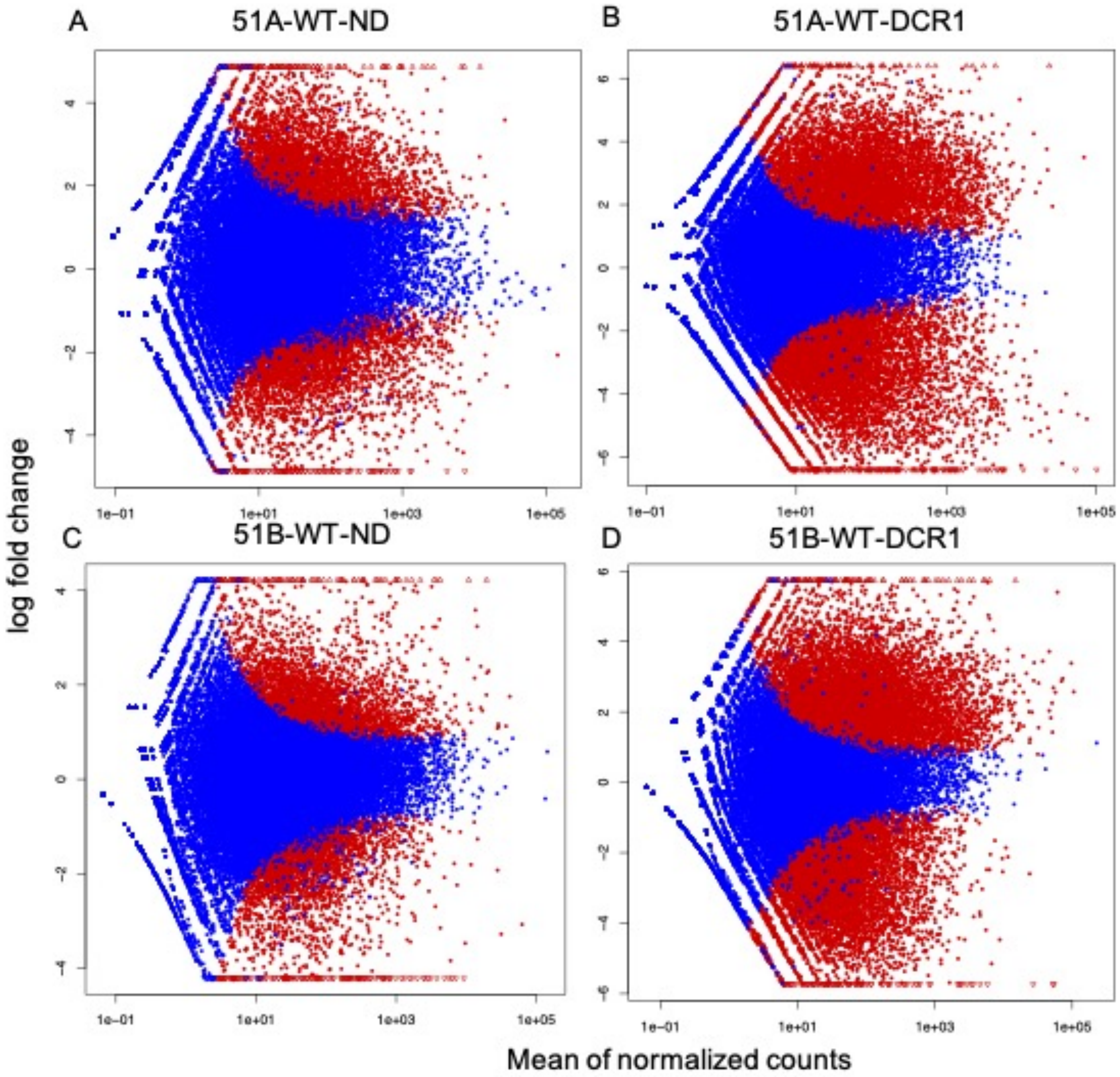

Supplementary Figure 2: MA plots showing the statistically significantly differentially expressed genes (in red) in each feeding sample against the wildtype serotype as identified by DESeq2.

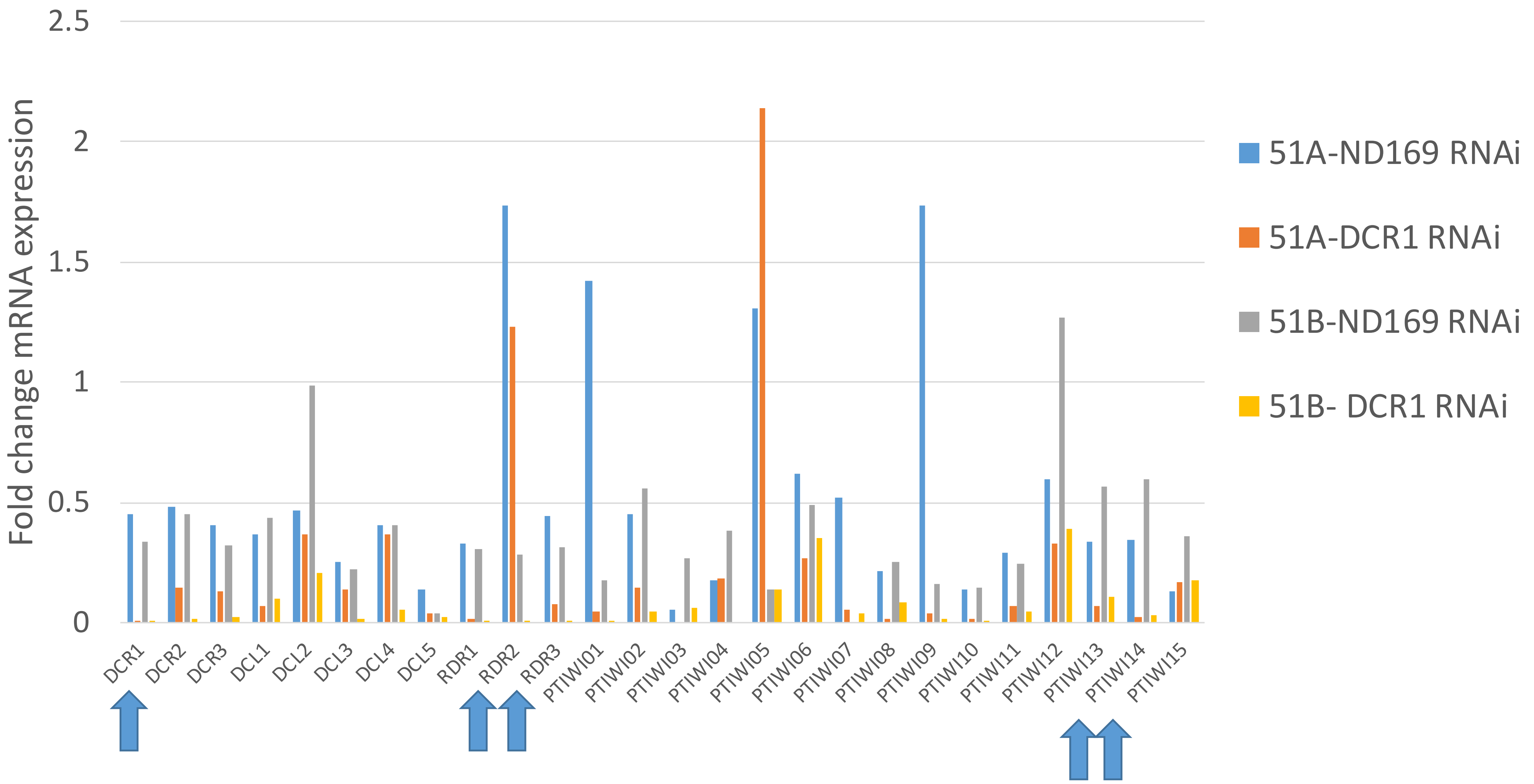

Supplementary Figure 3: Fold change of mRNA expression (y-axis, knockdown/wildtype) is shown for the RNAi components of *Paramecium tetraurelia* (Dicers, RNA dependent RNA polymerase (RDR), and Ptiwis). Blue arrows indicate RNAi components involved in the feeding pathway (DCR1, RDR1, RDR2, PTIWI12, PTIWI13).

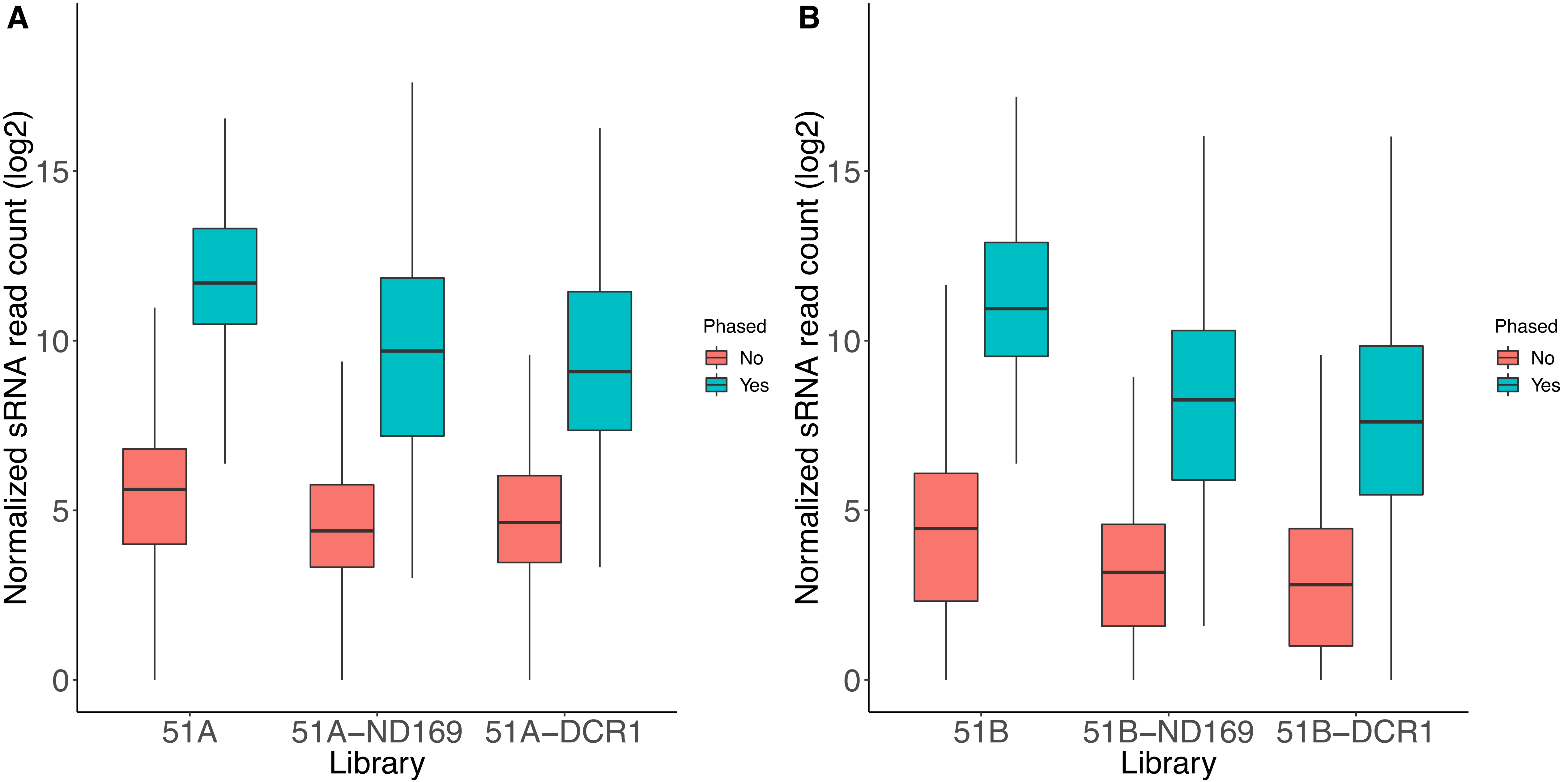

Supplementary Figure 4: A,B) Boxplots of the 1618 KDCS normalized endo-siRNA read counts (y-axis; log2) of serotype (51A, 51B respectively) and their knockdowns (ND, DCR1) categorized in to phased, and unphased loci.

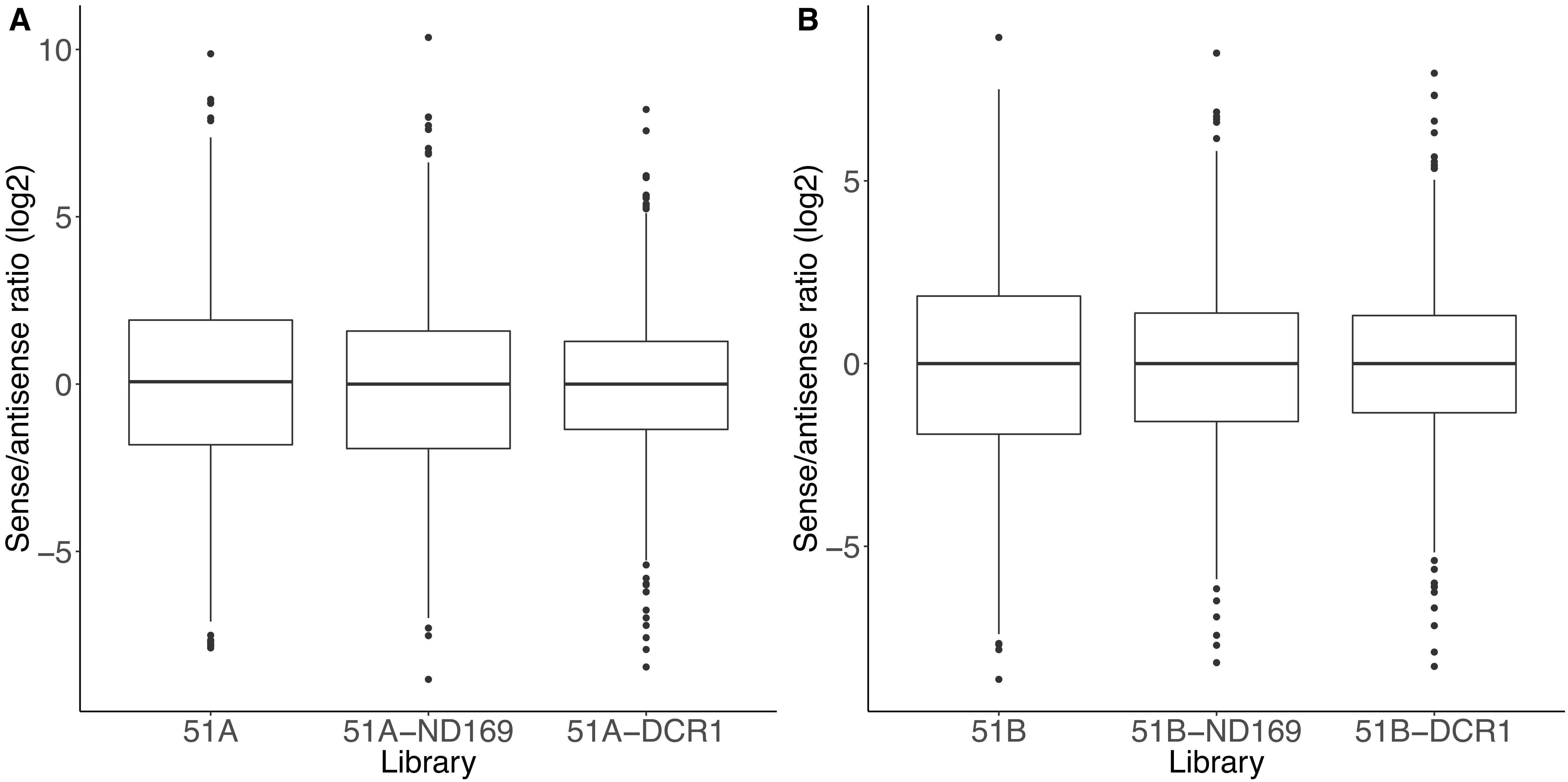

Supplementary Figure 5: A,B) For the different libraries (x-axis) the observed sense/antisense reads ratio (y-axis; log2) of the 1618 endo-siRNA clusters are shown.

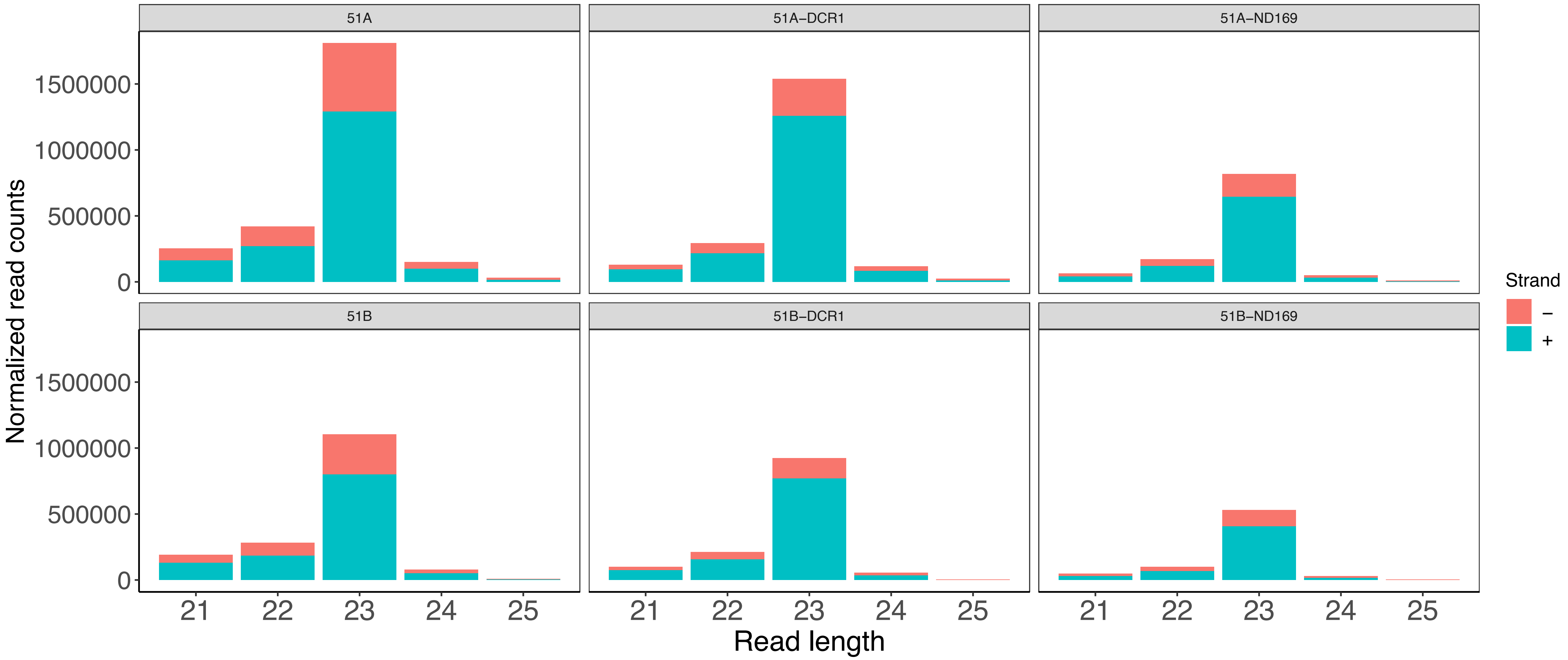

Supplementary Figure 6: Normalized read counts (y-axis) of the 1618 endo-siRNAs stratified in to different read lengths (x-axis) for the different libraries of serotypes 51A, and 51B are shown respectively.
